# Supplementary material for: Evaluation of a Wearable Non-Invasive Thermometer for Monitoring Ear Canal Temperature during Physically Demanding (Outdoor) Work
Source: Int J Environ Res Public Health. 2021 May 4;18(9):4896. doi: 10.3390/ijerph18094896 (PMC8125248; doi:10.3390/ijerph18094896)
Supplement: Supplementary file 1 [file ijerph-18-04896-s001.zip › Supporting Information files/Field study/Test protocol - part III Field study.pdf]

Test protocol for the in vivo validation and usability of wearable non-invasive thermometers

Manuscript: Evaluation of wearable non-invasive thermometer for monitoring inner-ear temperature of physically demanding occupations

Test protocol

Part I: Validation (lab study)

Part II: In vivo validation and usability in (lab study)

***Part III:* In vivo validation and usability (field study)**

Auteurs: C.C. Roossien [a*], A.P. Hodselmans [b], R. Heus [c], M.F. Reneman [a] and G.J. Verkerke [a,d]

[a] Department of Rehabilitation, University of Groningen, University Medical Center Groningen, Medicine, the Netherlands

[b] Center for Applied Research and Innovation in Health Care and in Nursing, Hanze University of Applied Sciences, the Netherlands

[c] Institute for Safety (IFV), the Netherlands

[d] Department of Biomedical Engineering University of Twente, the Netherlands

* Correspondence e-mail: c.c.roossien@umcg.nl

Keywords: Ambient conditions; continuous non-invasive thermometer; heat strain; heat stress; overheating; personal proactive clothing; physically active workers; thermal physiology.

Table of contents

1 Introduction 3
1.1 Background 3
1.2 Research motivation 3
1.3 Objective and aims 4
2 Materials and methods 5
2.1 Study design 5
2.2 Participants 5
2.2 Ethical considerations 5
2.3 Materials 6
2.3.1 Cosinussº 6
2.3.2 Ambient conditions box 6
2.3.3 Tympanic infrared thermometer 7
2.4 Steps 7
2.5 Data analysis 9
References 10

1 Introduction

1.1 Background

Heat strain among physically demanding occupations is of major concern and needs to be prevented. Heat strain is influenced by four environmental parameters [1]; ambient temperature (T_a) [2-4], air velocity, radiation and (relative) humidity (RH) [5-11]. These parameters can cause heat strain and stress due to a hampered loss of heat by the body which results in increased core temperature (T_c) [12]. Mainly two working conditions play an important role in gaining heat strain [1,5]. Firstly, working in (indoor and outdoor) hot (and humid) environments, as firefighters [3.13-15], underground mineworker [4,7,10] and workers in the steel industry [5], causes a hampered loss of heat by the body during physically active work due to the ambient conditions as T_a and RH and solar radiation [1-12]. Secondly, certain physically demanding occupations require workers to wear personal protective clothing (PPC) and equipment (PPE). This full-body clothing protects workers against chemical or biological substances, thermal exposure and mechanical impacts [16]. However, wearing PPC and PPE during the performance of physically active work, can cause heat strain due to thermal insulation (increase in T_a and RH) and evaporative resistance due to lack of air velocity in the PPC and/or PPE [1-2,5-7,12,17]. These two working conditions can cause heat strain resulting in health problems, such as exhaustion, dehydration, mental confusion and loss of consciousness, affecting productivity and risk perception [7-9,18]. In more extreme cases, heat strain can cause permanent damage and even be life-threatening [5,13,19-20]. Heat strain is influenced by individual factors [3,20-21] such as age, health, fitness and thermal comfort [4,7,10,12,16], resulting in increased metabolic rate, fatigue and health and safety problems [7,11-12,14,17,20,22-23].

1.2 Research motivation

By monitoring the T_c of workers and the ambient working conditions, heat strain could be prevented. T_c can be measured in several invasive and non-invasive ways [24]. Invasive measurements such as esophageal, rectal and gastrointestinal thermometers have high reliability [13,22-23], but are not suitable or inappropriate in a working situation [12,19,23,25-27]. Non-invasive methods, like skin and forehead thermometry, are nowadays wearable [28-29], but often impractical in a working situation because of interference with working conditions [25] or are unreliable [6,13,19,22,30]. Thus, presently, there is a lack of instruments available to continuously and unobtrusively monitor heat strain among physically active workers during the performance of their job [23,26,31-33]. To monitor and prevent heat strain in individual physically active workers, and for the sake of patient health, a reliable, non-invasive and continuous system of measuring in the form of a wearable thermometer is needed [23,31-32,34-37].

A new non-invasive sensor system, the CORTES^2 (Core Temperature and Environmental Sensor System) has been developed. This wearable thermometer measures tympanic temperature using an infrared (IR) sensor [31,38] positioned in the ear canal. Moreover, it also measures ambient conditions (T_a and RH) nearby the participants using a wearable chest box. A new commercially available system, the Cosinussº C-med (Cosinussº GmbH, München, Germany) has also become available. The wearable and non-invasive nature of the CORTES^2 and Cosinussº thermometers, and their ability to measure T_c continuously and on a daily basis, is innovative compared to available products that do not have the combination of these features. They could form the basis of a useful, non-invasive and low-level measuring system, which is easy to use and non-obstructive for the worker and do not hinder the workability. These products could be used in (scientific) research focusing on the development of heat

strain, measured in real-life situations during the performance of different types of physically demanding occupations, and to indicate potential ways of preventing heat strain more effectively and to improve the health and safety of physically active workers during the performance of their jobs.

Research into the validity of the Cosinussº One has shown a systematic difference of -1.5ºC compared to infrared tympanic temperature [31]. The validity and usability of the Cosinussº C-med and its interactiveness in working conditions are currently unknown but expected to be higher due to a more accurate sensor. In this study, both systems were studied in terms of (concurrent) validity and usability in a laboratory and a field study, and compared to tympanic IR thermometer [15,39-40]. The CORTES^2, Cosinuss C-med and tympanic IR thermometer are all based on tympanic temperature measurement and therefore expected to have comparable outcomes. To compare the outcomes of this study with the validity of the Cosinussº One [31], a tympanic IR thermometer will be selected as reference. In medical settings, tympanic temperature is the clinical standard used to monitor the core temperature of adult patients [14,41-43] due to its fast, non-invasive nature [12] and similarity (±0.2C) to rectal temperature measurements [15,42,44-47]. Besides, multiple studies have stated that tympanic infrared temperature is a reliable method for research purposes [13,44-45,48]. However, the accuracy and validity of tympanic infrared thermometry is questionable when not the real tympanic, but aural temperature is measured [24,49-50]. It is mostly the aural temperature which is measured. While tympanic infrared thermometry is not considered the scientific gold standard, its advantages are that it can be applied easily by the participants and that it is the current clinical gold standard method used when workers are expected to be suffering from excessive heat strain (overheated). So, for this in-vivo study a tympanic IR thermometer will be used as a reference.

1.3 Objective and aims

The objective of this study are to investigate the validity and usability of the CORTES^2 and Cosinuss C-med thermometers in a controlled lab and real-life working conditions. The aims are (1) to test the validity of the thermometers in controlled conditions; (2) to test validity and (3) explore the usability of the CORTES^2 and Cosinussº C-med thermometers for monitoring individual tympanic temperatures in a lab study; (4) to test validity and (5) explore the usability of the system to measure tympanic temperatures during the performance of physically demanding occupations, (6) in relation to the micro-climate ambient conditions (T_{cli} and RH) nearby the participant in a field study.

The study design contains three experiments: (I) validation of the thermometer is in a thermostatic water bath, (II) in vivo validation and usability explored in a lab study, (III) in vivo validation and usability explored in a field study. This document contains the test protocol part III In-vivo validation and usability (field study) to answer aims (4) to test validity and (5) explore the usability of the system to measure tympanic temperatures during the performance of physically demanding occupations, (6) in relation to the micro-climate ambient conditions (T_{cli} and RH) nearby the participant in a field study.

2 Materials and methods

2.1 Study design

Based on the findings and results of the lab study, the field study will only be performed with the Cosinussº. The field study contained three stages: (1) validation measurements; (2) performance of daily jobs; and (3) validation measurements. To test validity, in stages one and three the T_c of participants will be measured at rest with the Cosinussº C-med and tympanic IR. The T_c will be measured five times with a frequency of one measurement per minute, resulting in a five-minute measurement. The Cosinussº C-med will be calibrated individually using the second measurement of stage one with the tympanic IR. During stage two, the T_{cli} and RH were measured using a wearable ambient conditions box. The duration of stage two depended on the duration of the participant's task, lasting between 30 minutes and three hours.

Usability will be explored using the user interface design method AEIOU (*Activities Environments Interactions Objectives Users*) through researchers' observations and feedback from the participants. In this descriptive observational study, usability aspects were easy-to-use, positioning and adjustability, wear ability by all kind of users, stability and fixation, and comfort. This observation needs to be performed during the performance of their daily jobs (*Activities)* while wearing the Cosinussº C-med (*Object*) and their PPC and/or PPE. The *Environments* will be the four real-life working locations of the physically active workers (*Users*).

2.2 Participants

The inclusion criterion are that participants were between 18 and 67 years old (representing the European working population). The exclusion criteria included lung, cardio and/or vascular diseases, claustrophobia and problems with losing body heat (as by heat intolerances or difficulties with body thermoregulation due to problems with sweating).

Participants will be recruited by distributing flyers (see Appendix I) in four selected companies with different working situations: (1) chemical cleaners working in chemical-proof PPC with PPE; (2) mechanics working in a warm and humid factory; (3) firefighters working in PPC and with PPE; (4) neighborhood maintenance employees working outdoor in different weather conditions. The diversity in work-related tasks and working conditions between the four participant groups, as specified, should ensure a broad picture of their influences on the validity and usability of the wearable thermometer. At least 26 participants should be included with at least 7 participant per job category. The minimum sample size will be calculated with a power analysis (non-inferiority trail with a power of 95%, significance level of 0.05, acceptable difference of ±0.2ºC) for the field study based on the results of the lab study (n≥26 participants with n≥7 per job category).

2.2 Ethical considerations

This study will be carried out in accordance with "The Code of Ethics of the World Medical Association (Declaration of Helsinki) for experiments involving humans". The Medical Ethics Committee of the University Medical Center Groningen, the Netherlands, issued a waiver for this study, stating that it does not involve medical research under Dutch law (field study: M17.209969). The participants will to

be informed about the study by an information letter (see Appendix I) and a verbal explanation before the start of the study. All participants need to sign the informed consent before participating in this study.

2.3 Materials

The following materials are required:

- Cosinussº C-med
- CORTES²
- Ambient condition box
- Tympanic IR thermometer
- Personal protective clothing; gas mask
- Laptop for notation of data
- Phone with Cosinuss One app
- Timer
- 70% cleaning alcohol

2.3.1 Cosinussº

The Cosinussº type C-med (Cosinussº GmbH, München, Germany) is a core thermometer, which can be worn in and around the ear like a hearing aid (dimensions: 45x38x18 mm, 6.5 grams), as shown in **Fout! Verwijzingsbron niet gevonden.**. Temperature is measured with a contact sensor integrated into a sensor head, which is placed in the ear canal. Data is transported via Bluetooth Smart 4.0 and made visible with the Cosinussº One app. The accuracy of the Cosinussº C-med is ±0.1ºC, with a measurement range of 0 to 50ºC and a working temperature from -15 to 55ºC [51].

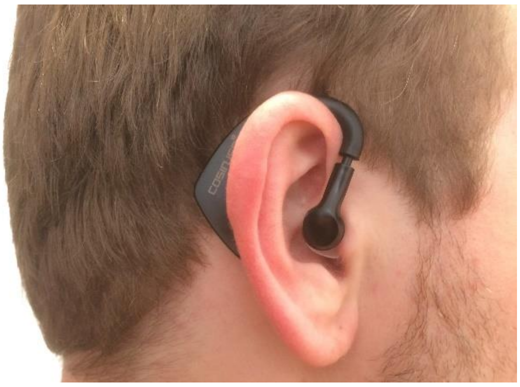

Figure 1: The wearable ear thermometer Cosinussº C-med.

2.3.2 Ambient conditions box

The ambient conditions box, worn with elastic chest belts (see **Fout! Verwijzingsbron niet gevonden.**), contains a temperature and humidity sensor (SHT15 Breakout, Sensirion, Staefa ZH, Switzerland). The box needs to be worn over the first layer of clothing, but under the PPC and PPE to measure the micro-climate nearby the skin of the participant inside the clothing (descripted as temperature inside clothing (T_{cli})) and RH and its effect of working activities on these conditions and its relation to the body

thermoregulation. The accuracy of the T_{cli} sensor is ±0.3ºC at 25ºC with a range of -40 to120ºC [53]. The absolute relative humidity accuracy is ±2% at 10 to 90% with a humidity range of 0 to 100% and a response time of 5 to 20 seconds [53]. The box will be validated in a climatic test cabinet (Clima Temperatur Systeme type C-40/350, Henchingen, Germany, with Pt 100 thermometers with an accuracy of T±0.3ºC and RH±1.5% [54]), resulting in a high to excellent correlation compared to the climatic test cabinet and Pt100 thermometer.

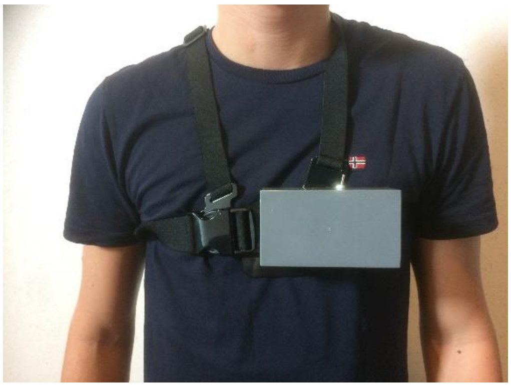

Figure 2: Ambient conditions box with temperature and humidity sensors and data receiver and storage.

2.3.3 Tympanic infrared thermometer

The Braun (Braun GmbH, Kornberg, Germany) ThermoScan® 7 type IRT 6520 tympanic infrared thermometer will be used as the reference thermometer [23,36]. This thermometer has an accuracy of ±0.2ºC within a body temperature range of 35–42ºC (RH 10–95%) [55]. This thermometer is referred to by validated in research of Purssell et al. (2009) as a reliable reference [31,56] for research about the core temperature of workers in hot environments [14-15,39] with a temperature change up to ±0.6ºC [45-46] in medical settings [42,44,57] and during exercise in heat [23,47,58]. All measurements with this tympanic IR thermometer were performed in offices with a constant room temperature of 20.0±2.0ºC and 45.0±5.0% humidity.

2.4 Steps

Preparation

1. Clean local validation location at company.
2. Put all the materials in position.
3. Check if the ambient conditions in validation environment are meeting the requirements (T_a=20.0±2.0ºC and RH=45.0±0.5%). If not, fix this problem.
4. Start Cosinussº and ambient conditions box by turning on and connect them. The Cosinuss needs to warm up for about 5 till 10 min.
5. Put new ear tip on tympanic IR thermometer.
6. Start laptop and open template for participants data.
7. Final check if everything is prepared for participant.

Introduce participant

8. Welcome participant.
9. Check if participant has received and read the information letter.
10. Inform participant about experiment by a verbal explanation.
11. Explain participant rights including its ability to stop at any moment and that the data will be progressed anonymous.
12. Check inclusion and exclusion criteria.
13. Ask if the participant understand everything and if there are any questions.
14. Ask participant to sign the informed consent. If the participant is not willing to sign the informed consent, the experiment will end here.

Validation (before working)

15. Put the Cosinussº in one ear of the participant and let it warm up for about 5 till 10 minutes until the output values are not fluctuating and constant.
16. Put tympanic IR thermometer in the other ear of the participant.
17. Measure every minute the temperature with the wearable thermometer and tympanic IR thermometer for five minutes.
18. Write down measurement output of Cosinussº and tympanic IR thermometer.
19. Remove wearable thermometer and tympanic IR thermometer.
20. Write down any comments of participant and observations about the Cosinussº and ambient condition box.
21. Store all the data.

Performance of daily job

22. Check connection between Cosinussº and ambient condition box.
23. Participant will perform its daily job for 30 up to three hours depending on participants task's or job description.
24. If possible, perform an observation by joining the participant during its performance of the job on safe distance. NOTE: wear PPC and PPE if required.
25. Write down any comments of participant and observations about the Cosinussº and ambient condition box.

Validation (after working)

26. Repeat step 15 up to including 21.

Finishing

27. Ask the participant for any (additional) input about the CORTES[2], Cosinussº and ambient condition box. Be aware of the usability aspects: easy-to-use, positioning and adjustability, wear ability by all kind of users, stability and fixation, and comfort.
28. Write down any comments of participant about the wearable thermometers.
29. Thank the participant for its participation.

Cleaning

30. Store all the data.
31. Cleaning the Cosinussº with 70% alcohol.

32. Remove ear tip and clean tympanic IR thermometer with 70% alcohol.
33. Cleaning the ambient condition box with 70% alcohol.
34. Clean and tidy up the rest of the materials and local validation location.

2.5 Data analysis

Calibration of the Cosinussº and CORTES^2 will be done using the standard clinical tympanic IR thermometer. During the in vivo validation measurements in rest, the T_c will be measured with the tympanic IR thermometer in combination with the Cosinussº or CORTES^2 thermometer in the other ear. The difference between the Cosinussº or CORTES^2 and the tympanic IR temperature will be considered as the calibration factor. In all cases, every second measurement will be selected to calculate the calibration factor (randomly chosen from the first three out of five in the field study).

For statistical analysis, IBM SPSS Statistics 25 needs to be used. For statistical analysis of the in vivo validation of the Cosinussº every fourth (out of five) measurement will be used in the field study (aim 4) (randomly chosen). Statistically significant differences will be studied with a paired t-test and an intraclass correlation coefficient (ICC, two-way random model, absolute agreement) will be calculated for normally distributed data. The ICC will be considered low when <0.39, moderate when 0.40-0.59, high when 0.60-0.79 and excellent when ≥0.80 [60]. Non-parametric data were also tested with the Wilcoxon signed rank test. P-values <0.05 were considered statistically significant. The Limits of Agreement (LoA) reflects the average differences between two different measurements and will be calculated as ±1.96*SDdifference [61]. The acceptable level of Limits of Agreement (LoA) will be 0.50. Bland-Altman plots were made of the individual difference between sessions against the individual mean of the two sessions, to analyze whether the magnitude of the difference will be related to the mean performance. A funnel shape indicates that the magnitude of the difference is related to the mean performance. Parameters were given for the t-tests together with their standard error of the mean. Sensitivity analysis will be performed to test differences between the fourth and fifth (field study) measurements, and all measurements. The usability of the lab and field study will be analyzed using descriptive statistics.

References

1. Gao C, Kuklane P, Ostergren P, Kjellstrom T. Occupational heat stress assessment and protective strategies in the context of climate change. International Journal of Biometeorology. 2018;62(3):359-371. doi: 10.1007/s00484-017-1352-y.
2. Fernandes A, Moreira D, Brito C, da Silva C, Sillero-Quitana M, Pimenta E, et al. Validity of inner canthus temperature recorded by infrared thermography as a non-invasive surrogate measure for core temperature at rest, during exercise and recovery. Journal of Thermal Biology. 2016;62(part A);50-55. doi:10.1016/j.jtherbio.2016.09.010.
3. Larsen B, Snow R, Vincent G, Tran J, Wolkow A, Aisbett B. Multiple Days of Heat Exposure on Firefighters' Work Performance and Physiology. PLoS ONE. 2015. doi:10.1371/journal.pone.0136413.
4. Anderson R, De Souza E. Heat stress management in underground mines. International Journal of Mining Science and Technology. 2017;27:651-655. doi:10.1016/j.ijmst.2017.05.020.
5. Krishnamurthy M, Ramalingam P, Perumal K, Kamalakannan L, Chinnaduai J, Shanmugam R, et al. Occupational Heat Stress Impacts on Health and Productivity in a Steel Industry in Southern India. Safety and Health at Work. 2017;8(1):99-104. doi:10.1016/j.shaw.2016.08.005.
6. Yang J, Weng W, Wang F, Song G. Integrating a human thermoregulatory model with a clothing model. Applied Ergonomics. 2017;61:168-177. doi:10.1016/j.apergo.2017.01.014.
7. Roghanchi P, Kocsis K. Challenges in Selecting an Appropriate Heat Stress Index to Protect Workers in Hot and Humid Underground Mines. Safety and Health at Work. 2018;9(1);10-16. doi:10.1016/j.shaw.2017.04.002.
8. Pijl J, Lundbye-Jensen J, Christiansen L, Ioannou L, Tsoutsoubi L, Dallas C, et al. High prevalence of hypohydration in occupations with heat stress - Perspectives for performance in combined cognitive and motor tasks. PLoS ONE. 2018. doi:10.1371/journal.pone.0205321.
9. Epstein Y, Moran D. Thermal effects on the Heat Stress Indices. Industrial Health. 2016;2016:388-398.
10. Sunkpal M, Roghanchi P, Kocsis K. A Method to Protect Mine Workers in Hot and Humid Environments. Safety and Health at Work. 2018;9(2):149-158. doi:10.1016/j.shaw.2017.06.011.
11. Dally M, Butler-Dawson J, Krisher L, Monaghan A, Weitzenkamp D, Sorensen C, et al. The impact of heat and impaired kidney function on productivity of Guatemalan sugarcane workers. PLoS ONE. 2018. doi:10.1371/journal.pone.0205181.
12. Jacklitsch B, Williams W, Musolin K, Coca A, Kim J, Turner N. NIOSH criteria for a recommended standard: occupational exposure to heat and hot. NIOSH, Cincinnati, OH, USA, Department of Health and Human Services, Centers for Disease Control and Prevention, National Institute for Occupational Safety and Health, DHHS.
13. Langridge P, Ruzic A, Larsen B, Lord C, Aisbett B. Assessing the validity of tympanic temperature to predict core temperature of firefighters in different environmental conditions, Proceedings of Bushfire CRC & AFAC 2012 Conference Research Forum. 2012:150-159.
14. Hemmatjo R, Motamedzade M, Aliabadi M, Kalatpour O, Farhadian M. The Effect of Various Hot Environments on Physiological Responses and Information Processing Performance Following Firefighting Activities in a Smoke-Diving Room. Safety and Health at Work. 2017;8(4):386-392. doi:10.1016/j.shaw.2017.02.003.
15. Calusic A, Marin M, Marija V, Branko P, Jelena M, Nevenka K, et al. Biomarkers of mild hyperthermia related to flashover training in firefighters. Journal of Thermal Biology. 2012;37(8):548-555. doi:10.1016/j.jtherbio.2012.06.007.

16. Zare M, Dehghan H, Yazdanirad S, Khoshakhlagh A. Comparison of the Impact of an Optimized Ice Cooling Vest and a Paraffin Cooling Vest on Physiological and Perceptual Strain. Safety and Health at Work. 2019;(in press). doi:10.1016/j.shaw.2019.01.004.
17. Costello J, Stewart K, Stewart I. The effects of metabolic work rate and ambient environment on philological tolerance times while wearing explosive and chemical personal protective equipment. BioMed Research International. 2015;2015:1-7. doi:10.1155/2015/857536.
18. Butts C, Smith C, Ganio M, McDermott B. Physiological and perceptual effects of a cooling garment during. Applied Ergonomics. 2017;59. doi:10.1016/j.apergo.2016.10.001.
19. Lim C, Byrne C, Lee J. Human Thermoregulation and Measurement of Body Temperature in Exercise and Clinical Settings. Ann Acad Med Singapore. 2008;37(4):347-353. doi:10.1109/ICSIMA.2013.6717956.
20. Morgado M, Talaia M, Teixeira L. A new simplified model for evaluating thermal environment and thermal sensation: An approach to avoid occupational disorders. International Journal of Industrial Ergonomics. 2017;60:3-13. doi:10.1016/j.ergon.2015.11.001.
21. Broday E, de Paula Xavier A, de Oliveira R. Comparative analysis of methods for determining the clothing surface temperature (tcl) in order to provide a balance between man and the environment. International Journal of Industrial Ergonomics. 2017;2017(57):80-87. doi:10.1016/j.ergon.2016.12.002.
22. Gonzalez-Alonso J, Christina T, Andersen S, Jensen F, Hyldig T, Nielsen B. Influence of body temperature on the development of fatigue during prolonged exercise in the heat. Hyperthermia and Fatigue. 1999:1032-1039.
23. Moran D, Mendal L. Core temperature measurement: methods and current insights. Sports Med. 2002;32(14):879-885. doi:10.2165/00007256-200232140-00001.
24. Taylor N, Tipton M, Kenny G. Considerations for the measurement of core, skin and mean body temperatures. Journal of Thermal Biology. 2014;46:72-101. doi:10.1016/j.therbio.2014.10.006.
25. Saurabh K, Rao H, Amrutur B, Sundarranjan A. Continuous core body temperature estimation via surface temperature measurements using wearable sensors: Is it feasible?. Biodevices (Conference paper). 2014:181-186.
26. McKenzie J, Osgood D. Validation of a new telemetric core temperature monitor. Journal of Thermal Biology. 2004;29(7-8):605-611. doi:10.1016/j.jtherbio.2004.08.020.
27. Guschlauer M, Maul A, Yan X, Herff H, Annecke T, Sterner-Kock A, et al. Zero-Heat-Flux Thermometry for Non-Invasive Measurement of Core Body Temperature in Pigs. PLoS ONE. 2016. doi:10.1371/journal.pone.0150759.
28. Martin-Vaquero J, Encinas A, Queiruga-Dios A, Bullon J, Martinez-Nova A, Gonzalez J, Bullon-Carbajo C. Review on Wearables to Monitor Foot Temperature in Diabetic Patients. Sensors. 2019;19(4):776. doi:10.3390/41904776.
29. van Marken Lichtenbelt W, Daanen H, Wouters L, Fonczek R, Raymann R, Severens N, van Someren E. Evaluation of wireless determination of skin temperature using iButtons. Physiology & Behavior. 2006;88(4-5):489-497. doi: 10.1016/j.physbeh.2006.04.026.
30. Ng K, Wong S, Lim S, Goh Z. Evaluation of the Cadi ThermoSENSOR Wireless Skin-Contact Thermometer Against Ear and Axillary Temperatures in Children. Journal of Pediatric Nursing. 2009; 5(3):176-186. doi:10.1016/j.pedn.2008.12.002.
31. Chaglla J, Celik N, Balanchandran W. Measurement of Core Body Temperature Using Graphene Inked Infrared Thermopile Sensor. Sensors. 2018;18(10):3315. doi:10.3390/s18103315.

32. Mazgoaker S, Ketko I, Yanovich R, Heled Y, Epstein Y. Measuring core body temperature with a non-invasive sensor. Journal of Thermal Biology. 2017;66:17-20. doi:10.1016/j.therbio.2017.03.007.
33. Pancardo P, Acosta F, Hernandez-Nolasco J, Wister M, Lopez-de-Ipina D. Real-Time Personalized Monitoring to Estimate Occupational Heat Stress in Ambient Assisted Working. Sensors. 2015;15(7):16956-16980. doi:10.3390/s150716956.
34. Uth M, Koch J, Sattler F. Body core temperature sensing: challenges and new sensor technologies. Procedia Engineering. 2016;168:89-92. doi:10.1016/j.proeng.2016.11.154.
35. Haines W, Momenroodaki P, Berry E, Fromandi M, Popovic Z. Wireless system for continuous monitoring of core body temperature. IEEE MTT-S International Microwave Symposium (IMS). 2017. doi:10.1109/MWSYM.2017.8058620.
36. Steck L, Sparrow E, Abraham J. Non-invasive measurement of the human core temperature. International Journal of Heat and Mass Transfer. 2011;54:975-982. doi:10.1016/j.ijheatmasstransfer.2010.09.042.
37. Yeoh W, Lee J, Lim H, Gan C, Liang W, Tan K. Re-visiting the tympanic membrane vicinity as core body temperature measurement site. PLoS ONE. 2017. doi: 10.1371/journal.pone.0174120.
38. Aryal A, Ghahramani A, Becerik-Gerber B. Monitoring fatigue in construction workers using physiological measurements. Automation in construction. 2017:154-165. doi:10.1016/j.autcon.2017.03.003.
39. Itani M, Ghadder N, Ouahrani D, Ghali K, Khater B. An optimal two-bout strategy with phase change material cooling vests to improve comfort in hot environment. Journal of Thermal Biology. 2018;72:10-25. doi:10.1016/j.jtherbio.2017.12.005.
40. Kofler P, Burtscher M, Heinrich D, Bottoni G, Caven B, Bechtold T, et al. Performance limitation and the role of core temperature when wearing light-weight workwear under moderate thermal conditions. Journal of Thermal Biology. 2015;47:83-90. doi:10.1016/j.jtherbio.2014.11.007.
41. Garcia-Souto M, Dabnichki P. Core and local skin temperature: 3e24 months old toddlers and comparison to adults. Building and Environment. 2016;104:286-295. doi:10.1016/j.buildenv.2016.04.016.
42. Kocoglue H, Goksu S, Isik M, Akturk Z, Bayazit Y. Infrared tympanic thermometer can accurately measure the body temperature in children in an emergency room setting. Int. J. Pediatr. Otorhinolaryngol. 2002;65(1):39-43. doi:10.1016/S0165-5876(02)00129-5.
43. Nederlands Huisartsen Genootschap. NHG-werkgroep Standaard Kinderen met koorts (derde herziening). Huisarts Wet, 2016;11:484-491.
44. van Staaij B, Rovers M, Schilder A, Hoes A. Accuracy and feasibility of daily infrared tympanic membrane temperature measurements in the identification of fever in children. Int. J. Pediatr. Otorhinolaryngol. 2003;67(10):1091-1097. doi:10.1016/S0165-5876(03)00198-8.
45. Gasim G, Musa I, Abdien M, Adam I. Accuracy of tympanic temperature measurement using an infrared tympanic membrane thermometer. BMC Res. Notes. 2013;6(194). doi:10.1186/1756-0500-6-194.
46. Purssell E, While A, Coomber B. Tympanic thermometry-normal temperature and reliability. Paediatr. Nurs. 2009;21(6):40-43. doi:10.7748/paed2009.07.21.6.40.c7151.
47. Moran-Naborra R, Courel-Ibanez J, Martinez-Cava A, Conesa-Ros E, Sanchez-Pay A. Validity of Skin, Oral and Tympanic Temperatures During Exercise in the Heat: Effects of Wind and Sweat. Annals of Biomedical Engineering. 2018;1-15. doi:10.1007/s10439-018-02115-x.

48. Coso J, Aguado-Jimenez R, Mora-Roderiguez R. Infrared tympanic thermometry in a hot environment. Int. J. Sports Med. 2008;29(9):713-718. doi:10.1055/s-2007-989417.
49. Casa D, Becker S, Ganio M, Brown C, Yeargin S, Roti M, et al. Validity of devices that assess body temperature during outdoor exercise in the heat. J. Athl. Train. 2007;42(3):333-342. doi:10.4085/1062-6050-44.2.124.
50. Ganio M, Brown C, Casa D, Becker S, Yeargin S, McDermott B, Boots L, et al. Validity and Reliability of Devices That Assess Body Temperature During Indoor Exercise in the Heat. J. Athl. Train. 2009;44(2):124-135. doi:10.4085/1062-6050-44.2.124.
51. Cosinussº. Technisches Datenblatt Cosinussº One. Cosinuss GmbH, Munchen, Germany. 2016.
52. Melexis N.V. Data Sheet MLX90614 family: Single and dual zone Infra Red Thermometer in TO-39. Melexis N.V., Ieper, Belgium. 2015.
53. Sensirion. Datasheet SHT1x (SHT10, SHT11, SHT15) - Humidity and Temperature Sensor. Sensirion AG, Staefa ZH, Switzerland. 2010.
54. CTS GmbH. CTS-Climatic Test Chambers. CTS GmbH, Hechingen, Germany. n.d.
55. Braun GmbH. ThermoScan® 7 met Age Precision® – IRT6520 - user manual. 30 April 2017. [Online]. Available: www.brauntherms.com/nl/producten/thermoscan-7-oor-thermometer-irt6520/.
56. Nadipi Reddy P, Cooper G, Weigthman A, Hodson-Tole E, Reeves N. Walking cadence affects rate of plantar foot temperature change but not final temperature in younger and older adults. Gait & Posture. 2017;52:272-279. doi:10.1016/j.gaitpost.2016.12.008.
57. Ammann R, Teuffel O, Agyeman P, Amport N, Leibundgut K. The Influence of Different Fever Definitions on the Rate of Fever in Neutropenia Diagnosed in Children with Cancer. PLoS ONE. 2015. doi:10.1371/journal.pone.0117528.
58. Ouahrani D, Itani M, Ghadder N, Ghali K, Khater B. Experimental study on using PCMs of different melting temperatures in one cooling vest to reduce its weight and improve comfort. Energy and Buildings. 2017;155:533-545. doi:10.1016/j.enbuild.2017.09.057.
59. Ansell Protective Solutions AB. Product sheet Trellechem® Super. Ansell Protective Solutions AB. Trelleborg, Sweden. 2017.
60. Cicchetti D. Guidelines, criteria, and rules of thumb for evaluating normed and standardized assessment instruments in psychology. Psychol. Assess. 1994:284-290.
61. Bland J, Altman D. Measuring agreement in method comparison studies. Stat. Methods Med. Res. 1999;8(2):135-160. doi:10.1177/096228029900800204.
